# Brefeldin A and Cytochalasin B reduce dengue virus replication in cell cultures but do not protect mice against viral challenge

**DOI:** 10.1099/acmi.0.000041

**Published:** 2019-07-22

**Authors:** Kleber Juvenal Silva Farias, Paula Renata Lima Machado, Renato Ferreira de Almeida Júnior, Benedito Antônio Lopes da Fonseca

**Affiliations:** ^1^ Department of Internal Medicine, School of Medicine of Ribeirao Preto – University of Sao Paulo, Avenida Bandeirantes, 3900, Monte Alegre, 14049-900, Ribeirao Preto SP, Brazil; ^2^ Program of Graduate Studies on Applied Microbiology and Immunology, School of Medicine of Ribeirao Preto – University of Sao Paulo, Avenida Bandeirantes, 3900, Monte Alegre, 14049-900, Ribeirão Preto SP, Brazil; ^3^ Department of Clinical Analysis and Toxicology, Federal University of Rio Grande do Norte, Street General Gustavo Cordeiro de Farias, 384, Petropolis, 59012-570 Natal, RN, Brazil

**Keywords:** DENV-2, Cell Culture, Mice, BFA, CB, Viral load monitoring, qRT-PCR

## Abstract

**Background:**

Dengue is an emerging arboviral disease caused by dengue virus (DENV). DENV belongs to the family *Flaviviridae* and genus *Flavivirus*. No specific anti-DENV drugs are currently available.

**Methods:**

We investigated the antiviral activity of Brefeldin A (BFA) and Cytochalasin B (CB) against this infection. The drugs BFA and CB were used in the *in vitro* treatment of dengue-2 virus (DENV-2) infections in Vero cell cultures and in protection from lethality by post-challenge administration in Swiss mice. Viral load was quantified by qRT-PCR and plaque assay in Vero cell cultures, post-infection, treated or not with the drugs. Post-challenge drug levels were evaluated by survival analysis.

**Results:**

Our results indicate that doses of 5 µg ml^−1^ of BFA and 10 µg ml^−1^ of CB are not toxic to the cells and induce a statistically significant inhibition of DENV-2 replication in Vero cells when compared to control. No BFA- or CB-treated mice survived the challenge with DENV-2.

**Conclusion:**

These data suggest that BFA and CB have an antiviral action against DENV-2 replication in Vero cell culture, but do not alter infected mice mortality.

## Introduction

There are a large number of viruses in the family *Flaviviridae* that are common in nature. These are responsible for causing serious diseases in humans and animals. The family is composed of three genera: *Flavivirus*, *Pestivirus* and *Hepacivirus*. The flavivirus genus contains more than 70 viruses transmitted by arthropods, including dengue virus (DENV), Japanese encephalitis virus, tick-borne encephalitis virus, West Nile virus and yellow fever virus, and are considered emerging and re-emerging pathogens. Within each genus, viruses can be subdivided into antigenic complexes [1, 2].

Dengue viruses are members of the family *Flaviviridae* and classified epidemiologically as arboviruses. There are four serotypes of the dengue virus (DENV-1, DENV-2, DENV-3 and DENV-4) and all can cause both the classical form of the disease and dengue haemorrhagic fever. Virulence is directly proportional to the intensity with which the virus multiplies in the body. Human target cells for dengue virus replication are those belonging to the monocyte–macrophage lineage [3].

DENV is mainly transmitted by *Aedes aegypti*. Only the female mosquito transmits the disease, as it seeks blood as food during the development of its eggs [4].

The dengue virion is a spherical particle, approximately 50 nm in diameter and consists of an icosahedral nucleocapsid and envelope [5]. The viral capsid involves a central nucleus of RNA forming the nucleocapsid. The envelope originates from the inner membrane of the endoplasmic reticulum (ER), when the viral particles arise from the lumen of the ER to the cytoplasm [6].

DENV is an enveloped, positive, ssRNA virus with genome of approximately 11 kb containing a single ORF. The ORF is processed by virally encoded protease (NS2B and NS3) to generate individual proteins: three structural proteins, capsid (C), pre-membrane/membrane (PrM/M) and envelope (E), and seven non-structural (NS) proteins, NS1, NS2A/2B, NS3, NS4A/4B and NS5, flanked by 5'- and 3'-non-translated regions (5'NTR/3'NTR). The structural proteins form the viral particle while the non-structural proteins participate in replication and invasion of the immune system [5, 7]

Dengue viruses are transmitted to humans by *Aedes aegypti* or *Aedes albopictus* mosquitoes. The virus can replicate in mononuclear cells (monocytes, macrophages and dendritic cells), in specific organs such as spleen, liver, lymph node, kidney and other organs [8]. Dengue viruses enter into the host cell by receptor-mediated endocytosis and replicate in the cytoplasm after 12–16 h of infection. The receptor molecules on the surface of the target cell that the DENV binds to in the initial phase of infection are unknown, although evidence suggests that heparan sulfate is an active participant at this stage of infection [9, 10]. After penetration into the cytoplasm, dissociation of E homodimers takes place as a result of the acidic environment in the endosome, resulting in fusion of the viral envelope with the endosomal membrane. After fusion has occurred, the nucleocapsid is released into the cytoplasm, where the capsid proteins and the RNA dissociate, initiating replication of the genomic uncoating RNA and assembly of the viral particle [7]. Viral RNA replicates in perinuclear areas, mediated by a negative-polar RNA that serves as a template for virus RNA replication. The viral RNA is translated into a single polyprotein that is cleaved co- and post-translationally by cellular and virus-derived proteases into three structural proteins and seven NS proteins [11]. The translation of RNA occurs in the ER. The NS proteins initiate viral genome replication at the intracellular membranes, resulting in the production of more viral RNA strands [12, 13]. Newly synthesized RNA is packed by C proteins to form the nucleocapsid. Non-infectious immature viral particles formed contain the glycoproteins E and prM, lipid membrane and nucleocapsid. Immature viral particles are transported to the Golgi complex where cleavage of prM by the furin (host protease) occurs by removal of the glycine portion of this protein, resulting in mature viral particles being released from the host cell by exocytosis of the actin filaments [14].

There are no antiviral DENV drugs available at present; supportive treatment focuses on fluid therapy and close clinical monitoring during the critical phase of dengue disease.

RNA viruses, like DENV, alter cellular membranes during their replication, and viral RNA synthesis occurs in membranous vesicles produced by the ER. During replication, viral capsid protein is responsible for recruitment of the viral genome during encapsidation, forming a nucleocapsid that springs into the ER lumen, acquiring membranes and the structural proteins E and prM [15]. The new viral particle migrates through the secretory pathway to be released by exocytosis. Brefeldin A (BFA) inhibits secretion and morphological changes in other organelles, leading to a dissociation of the Golgi complex, preventing the secretion of lipid vesicles [16, 17]. Previous studies with BFA have shown that lipid-containing viruses which mature along the secretory pathway are modified by BFA [18–20].

Studies by Mirazimi *et al*. [21] showed for the first time also viruses which assemble and remain in the ER are affected by BFA, which reduced progeny virus yield by 99 %. The BFA was used in a study by Iglesias *et al*. [22] in which they observed that this drug inhibited the high titre of dengue virus. Experiments with BFA showed that the virus needs the traffic vesicular of proteins for their replication, therefore the presence of the drug provoked an effect inhibitor in the replicative cycle of the Kunjin virus [23], as well as for the West Nile virus in Vero cells [24]. The inhibitory effect of BFA on virus replication in mammalian cells has already been studied with other viruses, such as human immunodeficiency virus (HIV-1), and the rubella virus [15, 25]. In the study realized by Tamura *et al*. [26] showed that BFA inhibited the replication of vaccinia virus and Newcastle disease virus. Study using BFA against encephalomyocarditis virus, demonstred that BFA antagonized the antiviral action of IFN by inhibiting IFN-induced enzymatic pathways [27].

The actin cytoskeleton is an important structure responsible for transport in cells, but it has a key role in stages of viral replication such as maturation and release. The actin cytoskeleton is also critical for clathrin-coated structure that is fundamental for flaviviruses that enter mammalian and mosquito cells by clathrin-dependent endocytosis [28]. Chu *et al*. [29] showed for the first time that actin filaments directly participated in the budding of West Nile virus at the plasma membrane. Perturbation of actin filaments by Cytochalasin B (CB) strongly inhibited the release of West Nile virus (~10 000-fold inhibition). Boulanger *et al*. [30] have also postulated that the disruption of actin filaments by cytochalasin could disengage the interaction between actin and its membrane linker, destabilizing the interaction between fowlpox virus precursors and the plasma membrane, and the virus yield was reduced. Bohn *et al*. [31] showed that measles virus-infected HeLa cells treated with CB had a reduced viral yield. Studies using cytochalasin D (CD) have also shown significant results: Wang *et al*. [32], when treating ECV304 cells infected with DENV-2 with CD, reported a reduction of the viral titres in the first hours of infection, again demonstrating that actin filament disturbance blocks viral release. Acosta *et al*. [33], studying CD on DENV-2-infected C6/36 cells, observed that the drug significantly reduced viral titres when cells were pretreated. Mosso *et al*. [34] also found that CD inhibited DENV infection by more than 80 % in C6/36 cells in treatment situations before or after infection. However, CB antiviral activity against Sindbis and vesicular stomatitis virus was not observed by Coombs *et al*. [35]. Griffins and Compans [36] noted that CB inhibited viral replication, but did not reduce the infectivity of influenza viruses or vesicular stomatitis viruses.

**Fig. 1.**
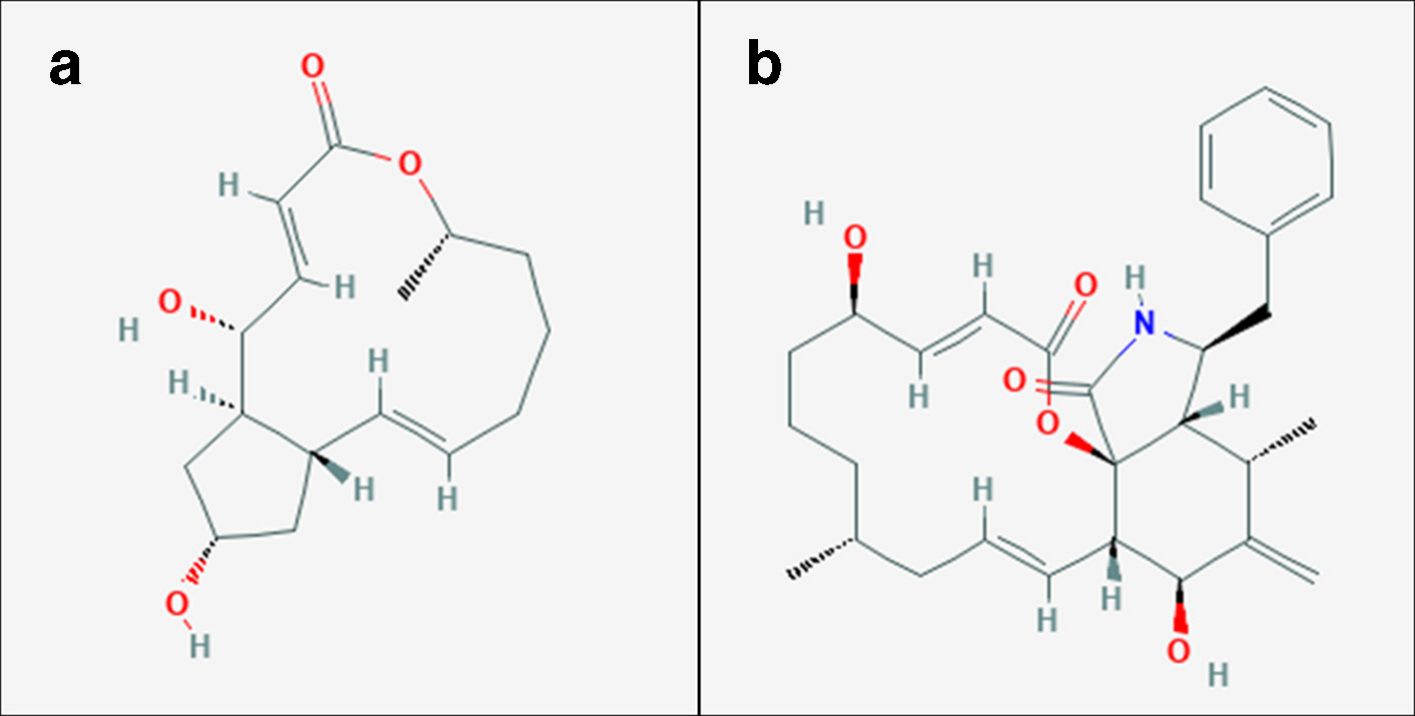
The two-dimensional chemical structure of brefeldin A (a) and cytochalasin B (b). Source: PubChem. URL: https://pubchem.ncbi.nlm.nih.gov.

BFA (Fig. 1a) and CB (Fig. 1b) exhibit various *in vitro* and *in vivo* biological actions, including antitumour, antifungal and antiviral activities with different mechanisms of action [26, 27, 35–37]. BFA, a unique fungal metabolite of a 13-membered lactone ring, blocks the secretion of proteins by dissociation of the Golgi apparatus, and CB, a mycogenic toxin known to disrupt the formation of actin polymers, destabilizes the cytoskeleton thus impeding the process of synthesis of cellular proteins [38, 39].

Thus, the aim of this study was to examine the *in vitro* and *in vivo* effects of BFA and CB on DENV-2 replication in DENV-2-infected Vero cells and DENV-2-infected mice, respectively. The results suggest that BFA and CB could be potential therapeutic agents to combat dengue infection.

## Methods

### Ethics statement

This study was carried out in strict accordance with the Brazilian Government’s ethical and animal experiment regulations. The procedures using animals in this research project were specified in accordance with the ethical principles established by the Brazilian College of Animal Experimentation (COBEA), and the experimental protocol was approved by the Committee on the Ethics of Animal Experiments of the University of Sao Paulo (CETEA/USP, Permit Protocol Number 080/2006). All surgical procedures were performed under ketamine/xylazine anaesthesia, and all efforts were made to minimize animal suffering.

### Cell cultures

Vero cells (continuous cell lineage originating from the kidney of African green monkeys) were grown in Leibovitz-15 (L-15) culture medium (Invitrogen) supplemented with 10 % FBS, 1 % l-glutamine 200 mM, 1 % penicillin G (100 IU ml^−1^) and streptomycin (100 µg ml^–1^). Vero cells were maintained at 37 °C. C6/36, an *Aedes albopictus* cell line, was cultured in L-15 medium supplemented with 10 % FBS, l-glutamine, 1 % penicillin G (100 U ml^−1^), and streptomycin (100 µg ml^−1^), at 28 °C.

### Virus stock

The parental DENV-2 New Guinea C strain used in this study was recovered from brains of intracerebrally infected 1- to 2-day-old suckling Swiss mice in the Virology Research Center, University of Sao Paulo, Ribeirao Preto, Brazil. The animals were killed, and the brains were removed in a sterile environment. Each brain was homogenized in 900 µl of bovine skin gelatin 4 % (Sigma Aldrich). The infected brain fluid (IBF) was centrifuged at 2800 ***g*** for 20 min at 4 °C. After centrifugation, the IBF was kept in ice to prevent further microbial loss, and then divided into aliquots and stored frozen at –80 °C. Virus stock supernatant fluid used in the present study was free of lipopolysaccharides and mycoplasmas.

### Dengue virus titration

Virus production was titrated by a plaque assay using Vero cells, as described by Russell and Nisalak [40]. Vero cells were seeded in a 12-well plate (6×10^5^ cells per well) in L-15 medium with 10 % FBS for 48 h at 37 °C. The medium was removed, and decimal serial dilutions of virus stock were added (0.1 ml per well) to the cells, which were then incubated for 2 h at 37 °C. Subsequently, L-15 medium containing 5 % FBS and 3 % carboxymethyl-cellulose (1 ml per well; overlay) was added, and the plate was incubated at 37 °C for 7 days. The overlay was removed on day 7, and cells were fixed with a solution of 10 % formaldehyde in PBS. After 2 h at room temperature, the formaldehyde solution was removed, and cells were washed twice with PBS and stained (15 min) with a 1 % crystal violet solution in 20 % ethanol. The plaques of cell lysis were counted, and the virus concentration was expressed as plaque-forming units (p.f.u.) per millilitre.

### Preparation of antiviral solutions

Antiviral solutions were prepared as follows: BFA was solubilized in ethanol (Santa Cruz Technology) at a concentration of 18×10^3^ µM (0.005 g/1 ml ethanol) and CB was solubilized in DMSO (Sigma) at a concentration of 2×10^3^ µM (0.001 g/1 ml DMSO). The DMSO or ethanol was injected through the rubber stopper of the vial to attain a final concentration of CB of 5 mg ml^−1^ and BFA of 1 mg ml^−1^, respectively. The drug suspensions were shaken until full dissolution. Stock solutions were stored at −20 °C. The drugs were diluted in L-15 medium to prepare working stock solutions immediately before use.

### Cytotoxicity test: MTT assay

To evaluate the cytotoxicity effect of the drugs (BFA and CB) and organic solvents (ethanol and DMSO) on the Vero cells, a 3-(4,5-dimethylthiazol-2-yl)−2,5-diphenyl tetrazolium bromide (MTT) colorimetric assay [41], which has been widely used and validated, was applied. The MTT was provided by Sigma-Aldrich. Vero cells were seeded in 96-well plates (1×10^4^ cells per well) and exposed to different concentrations of BFA (0, 0.05, 0.5, 5, 50 and 500 µg ml^−1^), CB (0, 0.01, 0.1, 1, 10, 100 and 1000 µg ml^−1^), ethanol (10, 5, 2.5, 1.2, 0.62, 0.31 and 0.15 %, v/v) and DMSO (10, 5, 2.5, 1.2, 0.62, 0.31 and 0.15 %, v/v); all compounds were diluted in L-15 medium. After treatment, the plates were incubated at 37 °C. A duration of 168 h was chosen for determining cytotoxicity *in vitro*. The assay was performed with the drugs added either only once or at 24 h intervals. After incubation, the supernatant was removed and 50 µl of L-15 containing MTT (1 mg ml^−1^) was added to each well. After 4 h of incubation at 37 °C, the supernatant was removed and 100 µl DMSO was added to each well to solubilize the formazan crystals. After shaking the plate, absorbance values obtained from cells treated with drugs, ethanol and DMSO were measured at 540 nm. Cell survival was determined by comparison between the absorbance values obtained from cells treated with drugs, ethanol and DMSO with untreated cells [41]. The percentage cytotoxicity of each dilution was calculated as (A/B)×100, where ‘A’ is the absorbance values from each treated well and B is the average of the controls. Viability was established from the average of triplicate determinations for each dilution. The 50 % cytotoxic concentration (CC_50_) was defined as the sample concentration that reduced cell viability by 50 % when compared to untreated controls. The 50 % inhibitor drug concentration of the viral effect (IC_50_) was derived from concentration–effect curves after linear regression analysis. The selective index (SI) was defined as the ratio of CC_50_ to IC_50_.

The calculation of the IC_50_ was performed under the conditions and concentrations described below.

### Time-of-addition studies

Monolayers of Vero cells seeded onto 24-well plates were infected with DENV-2 at an m.o.i. of 0.1. Time-of-addition studies were performed as follows. After-infection assay: after virus adsorption and washing, BFA (0, 0.05, 0.5 and 5 µg ml^−1^) or 1 % ethanol (control), CB (0, 0.01, 0.1, 1 and 10 µg ml^−1^) or 0.1 % DMSO (control) were added at different time points post-infection (pi) as follows: (i) 1 h after infection; (ii) 1 h after infection and at 24 h intervals for 7 days. DENV-2 replication of mock control was 0 µg ml^−1^. Infected cell supernatants were harvested at 0, 6, 12, 24, 48, 72, 96, 120, 144 and 168 h pi. Virus replication was monitored by quantitative real-time PCR and plaque assays. Each concentration of the drug was assayed in triplicate and repeated in two independent experiments and the results are shown as the mean values obtained from these experiments.

### Viral RNA extraction

RNA was extracted from 140 µl of each supernatant sample using the QIAamp Viral RNA kit (Qiagen) according to the manufacturer's directions.

### Real-time quantitative reverse transcription PCR (qRT-PCR) assay

In order to construct standard curves, the number of DENV-2 particles produced by infected cells was measured as the number of RNA copies quantified by qRT-PCR in serially diluted infected cell supernatants, after clearing by centrifugation. Each qRT-PCR contained 12.5 µl of the SYBR Green Master Mix reagent (Applied Biosystems), 0.5 µl RNase inhibitor, 0.13 µl Multiscribe (50 U µl^−1^), 0.5 µl primers (20 nM) DV2U (F 5′-AAGGTGAGATGAAGCTGTAGTCTC-3′) and DVL1 (R 5′-CATTCCATTTTCTGGCGTTCT-3′) specifically designed to anneal to the DENV-2 3'-untranslated region (3′-UTR), 5.87 µl diethyl pyrocarbonate (DEPC) water, and 5 µl RNA to a final volume of 25 µl [42]. The amplification protocol consisted of the following steps: 48 °C for 30 min and 95 °C for 10 min, followed by amplification with 40 cycles at 95 °C for 15 s and 60 °C for 1 min. The same protocol was used to quantify the DENV-2 RNA copies present in the supernatants of CB- or BFA-treated and untreated Vero cells infected with DENV-2, collected at defined post-infection intervals, as described above.

### Post-challenge protection

Forty 6-week-old females of wild-type young adult Swiss mice were infected intracerebrally with a dose of 100× LD_50_ (previously determined) of a neurovirulent DENV-2 in a volume of 100 litres (3×10^5^ p.f.u.) [43]. The protection of drugs was evaluated for 21 days. The animals used in the experiment were randomly divided into eight groups of five animals each. Thus, four groups were infected with DENV-2, and after 2 h two groups received either a single intraperitoneal (i.p.) dose of BFA (26.3 mg kg^−1^) [44] or CB (1.2 mg kg^−1^) [45]. Two groups received only PBS (mock) i.p. The other two groups received a single i.p. inoculum of either BFA or CB (negative control). Survival analysis was performed by observing these animals for the appearance of signs of disease such as tremors, hind limb paralysis, ruffled pile, muscle weakness and difficulty feeding. All mice were maintained under specific pathogen-free conditions.

The half-life of the drugs is short, which, after their i.p. or intravenous administration, are distributed in various organs within 15–180 min for CB and 60 min for BFA followed by a gradual reduction until their complete elimination after 24 h [46].

### Statistical analysis

Statistical analysis was used to assess the difference in viral yield, at time-defined intervals, by infected cells in contact with BFA or CB compared to control cells (without the drugs). Data were entered into the GraphPad Prism software, version 6.0 (GraphPad Software). In this study, our data are non-parametric and have two independent variables (time and treatment), so we used two-way ANOVA statistical tests followed by multiple Kruskal–Wallis comparisons. For all analyses, values of *P*<0.05 were considered statistically significant.

## Results

### Cytotoxicity of drugs in Vero cells

**Fig. 2.**
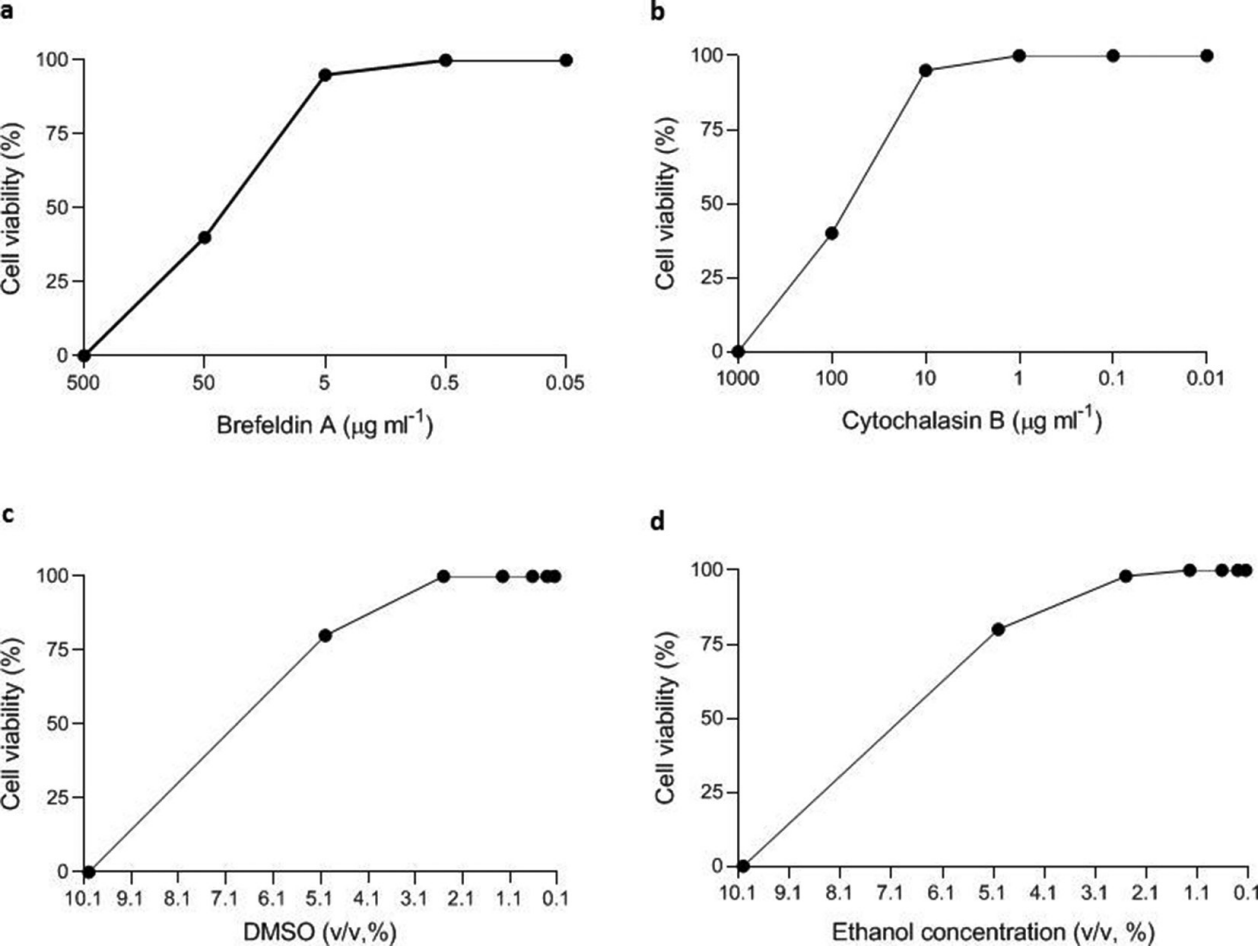
Cytotoxicity of the drugs (BFA and CB) and organic solvents (DMSO and ethanol) on Vero cells. BFA, brefeldin A; CB, cytochalasin B; DMSO, dimethyl sulfoxide.

Concentrations equal to or higher than 50 µg ml^−1^ (BFA) and 100 µg ml^−1^ (CB) were highly cytotoxic to Vero cells, while concentrations equal to or lower than 5 µg ml^−1^ (BFA) and 10 µg ml^−1^ (CB) did not induce significant cytotoxicity. Based on these data, concentrations equal to or lower than 5 µg ml^−1^ (BFA) and 10 µg ml^−1^ (CB) were used in the experiments (Fig. 2a, b). The final concentration of ethanol and DMSO in the antiviral solution stock of BFA and CB was 0.5 and 0.1 %, respectively. These concentrations were not cytotoxic to Vero cells. (Fig. 2c).

The CC_50_ values were 31.3 µg ml^−1^ for BFA and 43.5 µg ml^−1^ for CB.

### Effect of drugs on DENV-2 replication

**Fig. 3.**
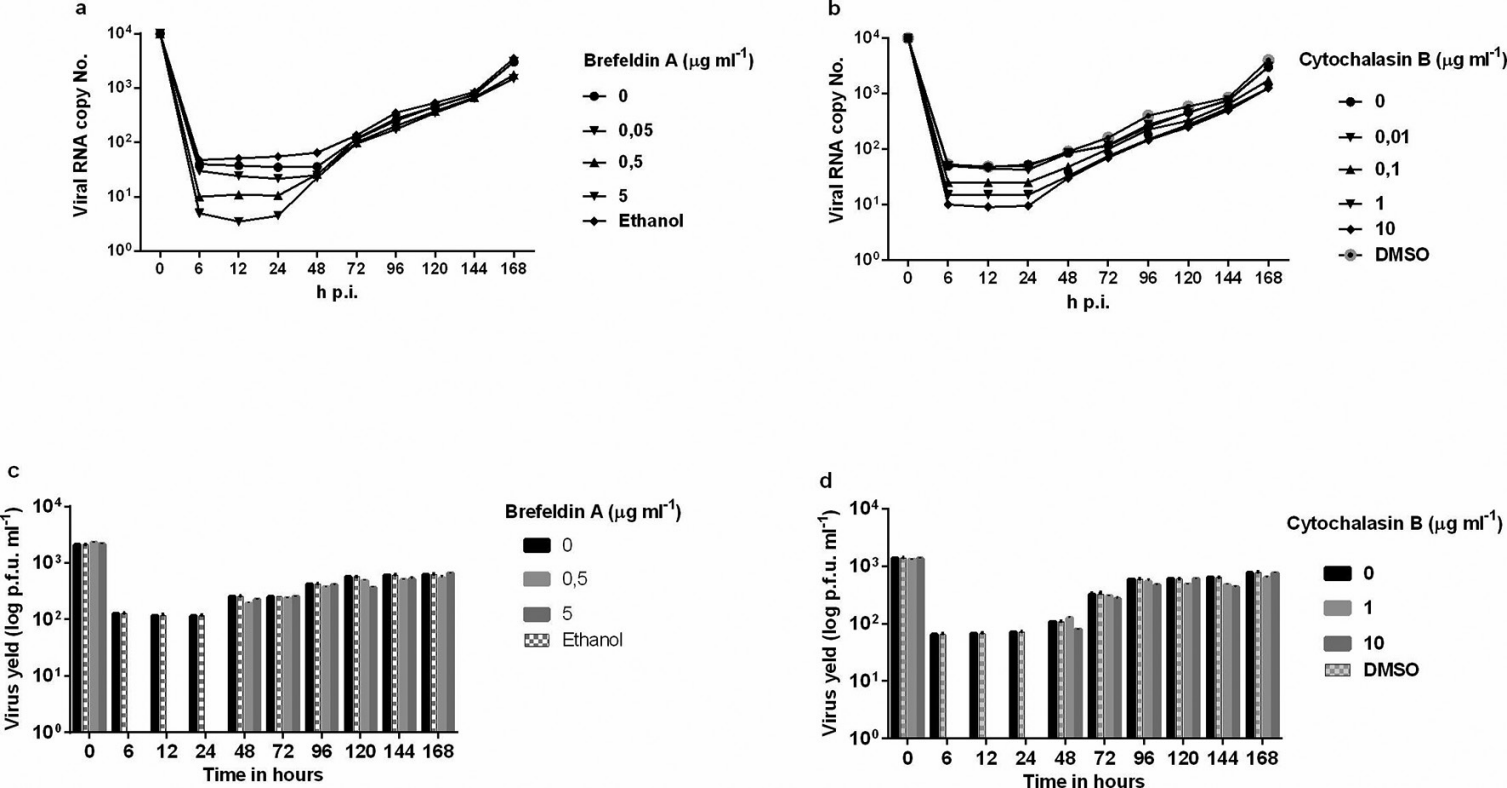
Action of BFA or CB, added 1 h post-infection, on DENV-2 replication in Vero cells. (a,b) The viral RNA present in the culture supernatants of Vero cells infected with DENV-2, treated or untreated with BFA or CB 1 h post-infection, was extracted and analysed by qRT-PCR. (c,d) Culture supernatants of Vero cells infected with DENV-2, treated or untreated with BFA or CB 1 h post-infection, were analysed by plaque assay. The results represent the average values of the viral RNA copy number (*P*<0.001). BFA, brefeldin A; CB, cytochalasin B; DENV-2, dengue-2 virus; qRT-PCR, real time quantitative reverse transcriptase PCR.

To determine whether BFA would inhibit DENV-2 replication, monolayers of Vero cells were infected with DENV-2 and incubated with 2 % FBS L-15 medium containing varying doses of BFA or CB. These drugs proved to be potent inhibitors of DENV-2, exhibiting antiviral activity, showing a dose-dependent inhibition of viral yields. Virus production by infected cells was detected by qRT-PCR using the standard curve to measure viral load and the results showed that BFA and CB reduced DENV-2 replication in Vero cells. Viral replication was inhibited by the addition of BFA at ≤5 µg ml^−1^ or CB at ≤10 µg ml^−1^ 1 h after infection when compared to untreated cells up to the first day after infection (Fig. 3a, b). Dilution (0.1 µg ml^−1^) of CB elicited partial inhibition (Fig. 3b). Further dilution (0.05 µg ml^−1^) of BFA or (0.01 µg ml^−1^) CB caused viral inhibition to disappear (Fig. 3a, b). The same results were obtained with the plaque assays; there was an excellent correlation between the plaque assays and the viral load determined by qRT-PCR (Fig. 3c, d).

**Fig. 4.**
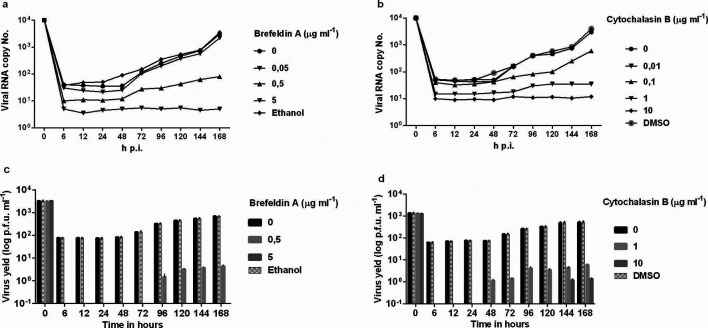
Action of BFA or CB, added at 24 h intervals, on DENV-2 replication in Vero cells. (a,b) The viral RNA present in the culture supernatants of Vero cells infected with DENV-2, treated or untreated with BFA or CB 1 h post-infection, and at 24 h intervals, was extracted and analysed by qRT-PCR. (c,d) Culture supernatants of Vero cells infected with DENV-2, treated or untreated with BFA or CB 1 h post-infection, and at 24 h intervals, were analysed by plaque assay. The results represent the average values of the viral RNA copy number (*P*<0.001). BFA, brefeldin A; CB, cytochalasin B; DENV-2, dengue-2 virus; qRT-PCR, real time quantitative reverse transcriptase PCR.

In an attempt to further improve inhibition of viral replication, BFA or CB was added 1 h after infection and at 24 h intervals after infection. The time-of-addition of the drugs after 1 h of infection and at 24 h intervals did not show differences in relation to when the drugs were applied only once (data not shown). When the number of viral RNA copies of DENV-2 was assayed in supernatants of Vero-infected cells treated with BFA or CB, we observed a reduction in viral yield compared to untreated cells (0 µg ml^−1^) up to the seventh day after infection (Fig. 4a, b). With this approach, both the plaque assay and qRT-PCR showed a statistically significant reduction in viral yield similar to that obtained for cells exposed to BFA or CB at 1 h after infection (Fig. 4c, d). We concluded that an appropriate amount of BFA or CB could inhibit DENV-2 replication without affecting cell viability.

The IC_50_ values were 2.72 µg ml^−1^ for BFA and 6.72 µg ml^−1^ for CB.

### Protection post-challenge

**Fig. 5.**
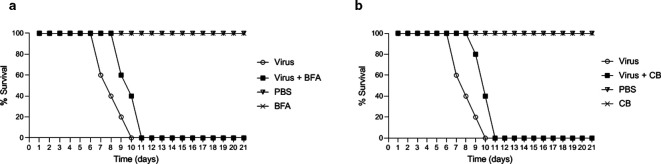
Survival curves of mice infected with DENV-2. (a,b) Swiss mice received BFA at 26.3 mg kg^−1^ or CB at 1.2 mg kg^−1^ 2 h post-infection. Survival analysis was performed by observing these animals for 21 days. Note that the mice of the positive control group died by the 10th day and those receiving BFA or CB and virus died on the 11th day. BFA, brefeldin A; CB, cytochalasin B; DENV-2, dengue-2 virus

All mice survived when only injected with drugs or PBS, while the animals that were inoculated with DENV-2 died on the 10th day after challenge. The groups of mice that received BFA or CB 2 h after viral infection showed 40 % survival on day 10, but all mice had died by day 11 (Fig. 5a, b).

## Discussion

Our results showed that BFA and CB reduced viral replication when the cells were treated at intervals of 24 h over 7 days of incubation.

Raekiansyah *et al*. [47] characterized an anti-DENV agent of fungus-derived BFA which can potentially be used as a lead compound for drug development of anti-DENV and other related viruses. To evaluate the antiviral activity of a sample in this study, Vero cells were infected and treated with BFA and incubated for 24 h when infected culture fluid (ICF) was harvested for virus quantification. In our study, the kinetics of ICF treated using both single dosing and multiple daily dosing of BFA for a period of 7 days showed good results when compared to single dosing. The study of Raekiansyah *et al*. [47] may have treated cultures of infected cells with multiple daily dosing for a extended period of time, and thus obtained better results.

In our *in vivo* experiments, the groups that received BFA or CB did not show satisfactory results, with percentage survival of DENV-2-infected mice treated or not with the drugs being similar.

The mechanism of disruption of vesicular transport between the ER and Golgi by BFA and destabilization of actin filaments by CB are critical steps for viral replication and release, and could provide a new tool to characterize the lifecycle of the virus. These drugs can also help researchers explore strategies for the development of DENV inhibitors. Furthermore, their antiviral activity can also be improved through derivative analyses or other approaches.

These results suggest that BFA inhibits DENV at an early phase in the viral replication cycle after viral entry. Our study proposes that BFA and CB are potential antiviral candidates against DENV-2 in culture cell. We speculate that BFA inhibits enveloped viruses, by blocking the trafficking of glycoprotein from the ER to Golgi, preventing the formation of new viruses, and that CB destabilizes actin filaments, thereby interfering with virus assembly and reducing the infection.
